# Telehealth adoption and use intentions among neurologists in Saudi Arabia: a cross-sectional study

**DOI:** 10.3389/fpubh.2026.1783573

**Published:** 2026-04-15

**Authors:** Naif H. Alanazi, Abdulaziz M. Alqarni, Fares Alzahrani

**Affiliations:** 1 Public Health Department, College of Health Science, Saudi Electronic University, Riyadh, Saudi Arabia; 2 Neurology Unit, Internal Medicine Department, Khamis Mushait General Hospital, Aseer Health Cluster, Ministry of Health, Khamis Mushait, Aseer, Saudi Arabia; 3 Department of Preventive Medicine, Armed Forces Hospitals Southern Region, Khamis Mushait, Aseer, Saudi Arabia

**Keywords:** intention to use telehealth, neurology, physician attitude, telehealth, teleneurology

## Abstract

**Background:**

Telehealth has increasingly been integrated into neurological practice worldwide, particularly following the COVID-19 pandemic. Despite national efforts to expand telehealth services in Saudi Arabia, evidence on neurologists’ telehealth use, attitudes, and intentions to adopt telehealth in routine practice remains limited.

**Methods:**

A national cross-sectional survey was conducted among neurologists practicing in Saudi Arabia to assess attitudes and intentions toward telehealth use. Participants were recruited via snowball sampling to complete a structured, self-administered questionnaire. The required sample size was calculated for a finite population and estimated at 281; a total of 300 neurologists were recruited to account for non-responses. Survey validity was established through expert content review and face validity testing, while reliability was confirmed using Cronbach’s alpha (0.73) and test–retest correlation (*r* = 0.82). Descriptive statistics summarized responses, and inferential analyses—including chi-square tests, Mann–Whitney U tests, and multivariate logistic regression, were performed to identify factors associated with intention to use telehealth, with *p* < 0.05 set as the criterion for statistical significance.

**Results:**

Overall, 78.7% (*n* = 236) intended to use telehealth. Intention was significantly higher among neurologists working in the Central region (39.8% vs. 20.3%, *p* = 0.017), governmental institutions (89.8% vs. 73.4%, *p* = 0.001), and tertiary care centers (51.3% vs. 32.8%, *p* = 0.030), and among consultants compared with specialists (36.0% vs. 21.9%, *p* = 0.006). Confidence in telehealth was higher for follow-up care than for new patients (82.7% vs. 24.0%, *p* < 0.001). The attitude toward telehealth was moderately positive (median 1.47, IQR = 0.47) and significantly higher among those intending to use telehealth (median 1.53 vs. 1.00, *p* < 0.001). Multivariate analysis showed that familiarity with telehealth (OR = 32.0, 95% CI: 8–126), positive attitudes toward follow-up care (OR = 21.0, 95% CI: 7–63), and institutional provision of audio services (OR = 4.84, 95% CI: 1.48–15) were strongly associated with the intention to use telehealth.

**Conclusion:**

Most surveyed neurologists reported a high intention to use telehealth. Confidence in telehealth was higher for follow-up care than for new-patient consultations. Overall attitudes toward telehealth were moderately positive and were significantly associated with the intention to use the service. Familiarity with telehealth, positive attitudes toward follow-up care, and institutional provision of audio services were identified as significant independent factors associated with telehealth adoption. These findings underscore the importance of individual and organizational factors in shaping neurologists’ willingness to adopt telehealth, suggesting that targeted strategies to enhance adoption should focus on increasing familiarity, promoting positive attitudes, and ensuring institutional support.

## Introduction

Telehealth has emerged as a transformative approach to healthcare delivery, enabling timely and efficient access to clinical services through digital technologies. In neurology, telehealth, often referred to as teleneurology, has gained increasing prominence due to its potential to address barriers to access and workforce shortages. During the COVID-19 pandemic, telehealth utilization expanded dramatically, with reported increases exceeding 600%, accelerating its integration into routine clinical practice worldwide (1). This rapid expansion has positioned telehealth as a core component of contemporary healthcare systems.

The World Health Organization (WHO) defines telemedicine as the delivery of healthcare services using information and communication technologies when distance is a limiting factor, supporting diagnosis, treatment, prevention, and continuing education (2). Although the terms telehealth and telemedicine are frequently used interchangeably, both emphasize remote patient care and have become essential tools for improving healthcare access, particularly in underserved and remote communities. In neurology, telehealth facilitates continuous patient education, longitudinal follow-up, and multidisciplinary collaboration, all of which are critical to the management of chronic and complex neurological disorders.

Despite growing global adoption, evidence on the utilization, acceptance, and perceived effectiveness of telehealth among neurologists remains limited. Existing studies suggest that teleneurology is feasible and accurate for selected neurological conditions; nevertheless, implementation patterns and acceptance differ considerably across healthcare systems (3). In Saudi Arabia, data describing the distribution of teleneurology services, neurologists’ attitudes, and their intention to use telehealth are scarce, highlighting a critical knowledge gap that needs to be addressed (4).

Saudi Arabia’s healthcare system is undergoing a substantial digital transformation aligned with Vision 2030, which aims to modernize the healthcare sector by expanding digital health services, improving the quality of care, and increasing access and efficiency across the health system. The Ministry of Health (MOH) prioritizes e-health initiatives to improve accessibility, efficiency, and quality of care. Since the early introduction of e-health strategies, the MOH has made significant progress in integrating telemedicine into healthcare delivery (5, 6). National platforms such as the *Seha* application and the 937- hotline consultation service illustrates the country’s intention to expand telehealth infrastructure, with utilization expected to increase further in the coming years (7). However, the successful implementation of teleneurology depends not only on technological readiness but also on healthcare workers’ engagement, regulatory frameworks, and adherence to patient safety and quality standards (8).

The need for teleneurology is further exacerbated by the global shortage of neurologists, particularly in low- and middle-income regions. In Arab countries, neurologist-to-population ratios have been estimated at approximately 1:300,000, limiting timely access to specialized neurological care. This indicates a substantial workforce shortage, which in turn limits timely access to specialized neurological services. The shortage is more likely to contribute to delays in diagnosis, limited follow-up capacity, and overall gaps in neurological care delivery across the region (9). Nevertheless, telehealth offers a pragmatic means of reducing or preventing these disparities by enabling remote consultations, strengthening interprofessional collaboration, and extending specialist services to underserved populations (10).

Teleneurology has demonstrated potential benefits across a range of neurological subspecialties, including outpatient consultations, inpatient services, and neurocritical care, while maintaining acceptable levels of clinical quality and patient satisfaction (3). Nevertheless, in Saudi Arabia, neurologists’ use of teleneurology, attitudes toward telehealth, and intention to adopt it remain insufficiently explored. In addition, concerns related to safety, confidentiality, cost, and physician–patient relationships warrant further investigation to support effective implementation.

Therefore, this study aimed to examine the distribution of teleneurology services in Saudi Arabia and to assess neurologists’ attitudes, intentions, and perceptions regarding telehealth usage in outpatient neurological care. The study also explored perceived applicability across neurological subspecialties, confidence in managing new and follow-up patients, and key barriers to adoption. Understanding the current state of teleneurology is essential for informing policymakers, healthcare leaders, and clinicians and for guiding the development of sustainable, high-quality telehealth services within the Saudi healthcare system.

## Materials and methods

### Study design

A national cross-sectional survey was conducted to assess the distribution, attitudes, and intentions regarding telehealth utilization among neurologists in Saudi Arabia beyond COVID-19-related restrictions. The study focused on teleneurology applications in outpatient neurological care across different healthcare settings and regions. The study adhered to the (Strengthening the Reporting of Observational Studies in Epidemiology) (STROBE) guidelines for cross-sectional studies.

### Sampling and recruitment

A non-probability snowball sampling technique was used to recruit neurologists practicing in Saudi Arabia. This approach was selected due to the absence of a centralized, publicly accessible national registry and the dispersed nature of the target population. Recruitment began with an initial group of neurologists (“seeds”) representing different geographic regions and professional levels. The survey link was disseminated electronically via professional and social networking platforms, including WhatsApp, Telegram, X (formerly Twitter), and the Saudi Neurology Society website. Participants were encouraged to share the survey within their professional networks until the target sample size of 300 participants was reached. While pragmatic, we acknowledge that this sampling method may introduce selection bias, as neurologists with a higher affinity for technology or digital communication may have been more likely to participate.

### Sample size calculation

The required sample size was calculated using the finite population correction formula. Assuming a 95% confidence level (*Z* = 1.96), a margin of error of 5%, and a conservative estimated prevalence of 50%, and based on an estimated population of 1,033 neurologists in Saudi Arabia (including consultants, specialists, and residents), the minimum required sample size was 281 participants. To compensate for potential non-responses and enhance statistical power, a target sample of 300 neurologists was set.

### Data collection instrument

Data were collected using a structured, self-administered online questionnaire adapted from previously validated instruments. The survey was informed by tools developed by Alqurashi et al. and Hmoud et al. and modified to address teleneurology practice in non-pandemic contexts (11, 12). Revisions were undertaken in consultation with neurologists experienced in telehealth to ensure contextual relevance. The questionnaire was administered in English, which is the standard language of medical education and practice in Saudi Arabia; therefore, translation into Arabic was not required.

The questionnaire was comprised of four sections:

1. Sociodemographic and professional characteristics (age, gender, region, institution type and care level, professional position, subspecialty).
2. Telehealth distribution and use, including availability and prior experience with teleneurology modalities.
3. Attitudes toward telehealth, covering use for new and follow-up patients, safety and privacy, quality of care, cost, and impact on the physician–patient relationship.
4. Intentions toward future telehealth use and preferred modalities.

### Validity and reliability

Content validity was established through expert review by a panel of neurologists, ensuring coverage of key domains relevant to teleneurology practice. Face validity was assessed through pilot testing with a small group of neurologists, which led to minor refinements to enhance clarity and relevance. Reliability testing demonstrated acceptable internal consistency (Cronbach’s alpha = 0.73). Test–retest reliability assessed over a two-week interval showed high stability (Pearson’s *r* = 0.82). The full questionnaire items are available in the Supplementary material.

### Statistical analysis

Data were analyzed using SPSS version 26 (IBM Corp., Chicago, IL, USA). Descriptive statistics were used to summarize participant characteristics and telehealth-related variables. Attitudinal responses were measured using a 3-point Likert scale (0 = Disagree/Negative, 1 = Neutral, 2 = Agree/Positive). Domain scores were calculated as the mean of the items within each domain, resulting in a composite score ranging from 0 to 2. These were analyzed as ordinal variables and reported as medians with interquartile ranges (IQRs).

Bivariate associations between participant characteristics and intention to use telehealth were examined using chi-square or Fisher’s exact tests, as appropriate. Differences in ordinal attitudinal scores were assessed using the Mann–Whitney U test. Multivariable analysis was conducted using binary logistic regression to identify independent factors associated with telehealth intention. Variables demonstrating statistical significance (*p* < 0.05) in the bivariate analysis were entered into the multivariate model (Enter method). Model fit was evaluated using the Hosmer–Lemeshow test, and statistical significance was set at *p* < 0.05.

### Ethical considerations

Ethical approval was obtained from the Institutional Review Board of the Ministry of Health, Saudi Arabia (IRB log No. 24–15 M; February 2024). Participation was voluntary, and informed consent was obtained electronically prior to survey administration. No identifiable personal information was collected. All data were stored securely and analyzed in aggregate form to ensure confidentiality.

## Results

### Participant characteristics

A total of 300 neurologists practicing in Saudi Arabia completed the survey. Most participants were aged 22–30 years (42.3%), followed by 31–40 years (37.0%), 41–50 years (18.3%), and >50 years (2.3%). The sample included 161 males (53.7%) and 139 females (46.3%). Participants were geographically distributed across the Central (35.7%), Western (30.3%), Eastern (24.0%), Southern (6.7%), and Northern regions (3.3%).

The majority of respondents were employed in governmental institutions (86.3%), with the remainder working in the private sector (13.7%). Nearly half practiced in secondary care settings (48.3%), followed by tertiary (47.3%) and primary care (4.3%). Current professional roles included consultants (33.0%), senior residents (24.3%), junior residents (21.3%), specialists (18.0%), and fellows (3.3%). Most neurologists reported ≤5 years of experience (58.0%). General neurology was the most common subspecialty (64.0%), followed by neurovascular/stroke (8.7%), epilepsy (7.3%), pediatric neurology (7.3%), and neuroimmunology (5.0%). Refer to Table 1 for more details.

**Table 1:** Sociodemographic characteristics of neurologists by intention to use telehealth (*N* = 300).

Variable	Total *n* (%)	Intention *n* (%)	No intention *n* (%)	*p-*value
Age group (years)				
22–30	127 (42.3)	102 (43.2)	25 (39.1)	0.31
31–40	111 (37.0)	84 (35.6)	27 (42.2)	
41–50	55 (18.3)	46 (19.5)	9 (14.1)	
>50	7 (2.3)	4 (1.7)	3 (4.7)	
Gender				
Female	139 (46.3)	105 (44.5)	34 (53.1)	0.21
Male	161 (53.7)	131 (55.5)	30 (46.9)	
Region				
North	10 (3.3)	5 (2.1)	5 (7.8)	0.017*
South	20 (6.7)	15 (6.4)	5 (7.8)	
Eastern	72 (24.0)	55 (23.3)	17 (26.6)	
Western	91 (30.3)	67 (28.4)	24 (37.5)	
Central	107 (35.7)	94 (39.8)	13 (20.3)	
Institution type				
Private	41 (13.7)	24 (10.2)	17 (26.6)	0.001*
Governmental	259 (86.3)	212 (89.8)	47 (73.4)	
Level of care				
Primary	13 (4.3)	9 (3.8)	4 (6.3)	0.030*
Secondary	145 (48.3)	106 (44.9)	39 (60.9)	
Tertiary	142 (47.3)	121 (51.3)	21 (32.8)	
Professional position				
Junior resident	64 (21.3)	49 (20.8)	15 (23.4)	0.006*
Senior resident	73 (24.3)	60 (25.4)	13 (20.3)	
Specialist	54 (18.0)	33 (14.0)	21 (32.8)	
Fellow	10 (3.3)	9 (3.8)	1 (1.6)	
Consultant	99 (33.0)	85 (36.0)	14 (21.9)	
Years of experience				
0–5	174 (58.0)	138 (58.5)	36 (56.3)	0.12
6–10	66 (22.0)	49 (20.8)	17 (26.6)	
11–15	47 (15.7)	39 (16.5)	8 (12.5)	
16–20	10 (3.3)	9 (3.8)	1 (1.6)	
>20	3 (1.0)	1 (0.4)	2 (3.1)	
Subspecialty				
General neurology	192 (64.0)	148 (62.7)	44 (68.8)	0.57
Pediatric neurology	22 (7.3)	19 (8.1)	3 (4.7)	
Neuroimmunology	15 (5.0)	15 (6.4)	0 (0.0)	
Neurovascular/stroke	26 (8.7)	20 (8.5)	6 (9.4)	
Epilepsy	22 (7.3)	16 (6.8)	6 (9.4)	

*Significant at *p* < 0.05.

### Availability, use, and preferences for telehealth

Nearly half of the participants (46.0%) reported that their institutions did not provide any telehealth services. The lack of institutional telehealth availability was significantly more common among neurologists who did not intend to use telehealth than among those who did (62.5% vs. 41.5%; *p =* 0.022). Institutions offering telephone/audio or video-based services were more frequently reported by neurologists who intended to use telehealth. Moreover, the majority of the respondents (54.7%) had not previously used telehealth services, with no significant difference by intention status. However, prior use of telephone/audio or video-based telehealth was more common among those intending to use telehealth (*p* = 0.001). In terms of preferred modes of communication, online video was the most common telehealth access method (61.7%), and it was significantly more common among neurologists who intended to use telehealth (66.5% vs. 43.8%; *p* = 0.001).

### Perceived applicability across neurology subspecialties

The total number of responses reflects neurologists’ overall perceptions of telehealth applicability across neurological subspecialties, with multiple responses permitted. A total of 637 responses indicated perceived applicability for new patients, while 1,542 responses were reported for follow-up patients, as shown in Table 2. Telehealth was perceived as most applicable in general neurology, epilepsy, headache, and pediatric neurology, particularly for follow-up care. Applicability was consistently higher among follow-up patients than among new patients across all subspecialties. Telehealth was perceived as least applicable to neurocritical care, neuro-ophthalmology, and other specialized clinics, with <2% of respondents endorsing its applicability to new patients.

**Table 2:** Neurologists’ perception of telehealth applicability across neurological specialties.

Neurological specialty/disorders	Telehealth applicability	
	For new patients, *n* (%)	For follow-up patients, *n* (%)
General neurology	222 (34.85)	271 (17.57)
Pediatric neurology	47 (7.38)	138 (8.95)
Epilepsy	101 (15.86)	215 (13.94)
Neurovascular/Stroke	32 (5.02)	133 (8.62)
Headache	84 (13.19)	180 (11.67)
Memory/Neurobehavioral disorder	42 (6.59)	97 (6.29)
Movement disorder	23 (3.61)	93 (6.03)
Neuromuscular disorder	11 (1.73)	112 (7.26)
Neuroimmunology	23 (3.61)	142 (9.21)
Neurocritical care	3 (0.47)	19 (1.23)
Neuro-ophthalmology	4 (0.63)	25 (1.62)
Neuro-infectious disease	16 (2.51)	56 (3.63)
Neuro-oncology	22 (3.45)	61 (3.96)
Other*	7 (1.10)	0 (0.00)
Total	637 (100.00)	1,542 (100.00)

Percentages were calculated based on the total number of responses for each category (new patients & follow-up patients).

Multiple responses were allowed. *Other included screening and development clinics and referral clinics.

### Familiarity and confidence with telehealth

Familiarity with telehealth technology emerged as the strongest predictor of intention to use telehealth (OR = 32.0). Over half of neurologists (53.3%) reported familiarity with telehealth technology, with significantly higher familiarity among those intending to use it (61.4% vs. 23.4%; *p < 0*.001). Furthermore, confidence in using telehealth was higher for follow-up patients (82.7%) than for new patients (24.0%). Neurologists who intended to use telehealth were significantly more confident in both new patient evaluations (30.1% vs. 1.6%; *p < 0*.001) and follow-up care (93.6% vs. 42.2%; *p* < 0.001). More details on telehealth availability, use, and preferences are provided in Table 3.

**Table 3:** Distribution and intention of using telehealth among neurologists in Saudi Arabia (*n* = 300).

Variable	Total *n* (%)	Intention *n* (%)	No intention *n* (%)	*p*-value
Telehealth services availability				
None	138 (46.0)	98 (41.5)	40 (62.5)	0.022*
Telephone/audio	83 (27.7)	77 (32.6)	6 (9.4)	
Online video	10 (3.3)	9 (3.8)	1 (1.6)	
Video + audio	69 (23.0)	52 (22.0)	17 (26.6)	
Telehealth services previously used				
None	164 (54.7)	129 (54.7)	35 (54.7)	0.001*
Telephone/audio	77 (25.7)	66 (28.0)	11 (17.2)	
Online video	12 (4.0)	11 (4.7)	1 (1.6)	
Video + audio	47 (15.7)	30 (12.7)	17 (26.6)	
Preferred mode of telehealth access				
Telephone/audio	115 (38.3)	79 (33.5)	36 (56.3)	0.001*
Online video	185 (61.7)	157 (66.5)	28 (43.8)	
Familiarity with telehealth technology				
Disagree	26 (8.7)	9 (3.8)	17 (26.6)	<0.001*
Neutral	114 (38.0)	82 (34.7)	32 (50.0)	
Agree	160 (53.3)	145 (61.4)	15 (23.4)	
Confidence in using telehealth for new patients				
Disagree	89 (29.7)	57 (24.2)	32 (50.0)	<0.001*
Neutral	139 (46.3)	108 (45.8)	31 (48.4)	
Agree	72 (24.0)	71 (30.1)	1 (1.6)	
Confidence in using telehealth for follow-up patients				
Disagree	9 (3.0)	3 (1.3)	6 (9.4)	<0.001*
Neutral	43 (14.3)	12 (5.1)	31 (48.4)	
Agree	248 (82.7)	221 (93.6)	27 (42.2)	

Values are presented as *n* (%). *p* values were calculated using the chi-square test. *p < 0.05 indicates statistical significance.

### Perceived barriers to telehealth adoption

From patient perspectives, acceptance of telehealth was the most frequently reported barrier to telehealth implementation (47.4%). In addition, limited patient knowledge of telehealth use (34.7%) was also commonly reported. From the physician’s perspective, suboptimal training and awareness (40.2%), provider acceptance (31.7%), and lack of confidence (28.1%) were the most cited barriers. At the healthcare system level, policy and quality approval requirements (38.2%), equipment demands (27.0%), and staff shortages, including IT support (27.0%), were identified as key limitations. Table 4 provides additional details on perceived barriers to telehealth adoption. This finding aligns with previous research, indicating that patient awareness, digital literacy, and trust in telemedicine platforms are essential determinants of telehealth success. Similarly, a lack of physician training was frequently reported as a barrier, reinforcing the need for structured training programs to improve clinicians’ confidence and competence in telehealth delivery.

**Table 4:** Neurologists’ perception of telehealth applicability across neurological specialties.

Perspective	Barrier	*n*	%
Patient-related	Increased costs for patients	26	5.47
	Patient acceptance of telehealth services	225	47.37
	Limited patient knowledge of telehealth use	165	34.74
	Patient privacy concerns	59	12.42
	Subtotal	475	100.00
Healthcare provider–related	Physician/provider acceptance	139	31.73
	Suboptimal training and awareness	176	40.18
	Lack of confidence in telehealth use	123	28.08
	Subtotal	438	100.00
Healthcare system–related	Policies and quality requirements before approval	219	38.15
	Increased costs for hospitals	42	7.32
	Equipment requirements (e.g., internet, devices)	155	27.00
	Shortage of staff (including IT expertise)	155	27.00
	Shortage of physicians	3	0.52
	Subtotal	574	100.00

Percentages were calculated within each perspective category. Multiple responses were permitted.

### Attitudes toward telehealth among neurologists

This study also investigated neurologists’ attitudes toward telehealth, which are summarized in Table 5. Overall, the participants’ attitudes were moderately positive, with a median score of 1.47 (IQR, 0.47) on a scale in which 2 represents positive agreement. Neurologists who reported an intention to use telehealth showed significantly more positive attitudes than those without such intention (median 1.53 (IQR, 0.40) vs. 1.00 (IQR, 0.44), *p <* 0.0001).

**Table 5:** Attitudes toward telehealth among neurologists in Saudi Arabia.

Aspect	Total (*n* = 300) Median (IQR)	Intention to apply telehealth		*P* value
		Yes (*n* = 236) Median (IQR)	No (*n* = 64) Median (IQR)	
Overall attitude	1.47 (0.47)	1.53 (0.40)	1.00 (0.44)	<0.0001
Use for new patients	1.25 (0.75)	1.25 (0.75)	0.75 (0.75)	<0.0001
Use for follow-up patients	2.00 (0.50)	2.00 (0.25)	1.25 (0.50)	<0.0001
Safety and privacy	1.33 (0.67)	1.33 (0.67)	1.00 (1.00)	<0.0001
Cost reduction	2.00 (0.50)	2.00 (0.25)	1.50 (1.00)	<0.0001
Patient–physician relationship	1.50 (0.50)	1.50 (0.50)	1.00 (1.00)	<0.0001

Values are presented as median (IQR). Attitude scores were derived from a 3-point ordinal scale (0 = Disagree/Negative, 1 = Neutral, 2 = Agree/Positive). The reported values represent the median of the mean domain scores. Comparisons were performed using the Mann–Whitney U test. *p* < 0.05 was considered statistically significant.

Regarding the use of telehealth for new patients, the overall median attitude score was 1.25 (IQR, 0.75). A similar median was found among neurologists intending to use telehealth, whereas those not intending to use telehealth reported significantly less favorable attitudes (median 0.75 (IQR, 0.75); *p* < 0.0001). In contrast, attitudes toward telehealth for follow-up patients were more favorable, with a median score of 2.00 (IQR, 0.50) overall among neurologists intending to use telehealth. Neurologists without intention to use telehealth reported significantly lower scores (median 1.25 (IQR, 0.50); *p* < 0.0001).

Perceptions related to safety and privacy showed an overall median score of 1.33 (IQR, 0.67) among neurologists intending to use telehealth, whereas those not intending to use telehealth reported lower scores [median 1.00 (IQR, 1.00); *p* < 0.0001]. Similarly, perceived cost reduction was rated positively [median 2.00 (IQR, 0.50)], with significantly lower scores among neurologists without intention to use telehealth [median 1.50 (IQR, 1.00); *p* < 0.0001]. Moreover, attitudes toward the impact of telehealth on the patient–physician relationship were moderately positive [median 1.50 (IQR, 0.50)] among neurologists intending to use telehealth, but significantly less favorable among those without such intention (median 1.00 (IQR, 1.00); *p* < 0.0001).

### Intention to use telehealth

Overall, 78.7% of neurologists (*n* = 236) reported an intention to use telehealth in their clinical practice, indicating a strong level of professional readiness for the adoption of teleneurology in Saudi Arabia. This level of acceptance is consistent with previous international research demonstrating an increase in physicians’ willingness to integrate telehealth following the COVID-19 pandemic. However, intention to use telehealth varies significantly by region, institution type, level of care, and professional role. Neurologists from the Central region were more likely to intend to use telehealth compared with those who did not (39.8% vs. 20.3%; *p* = 0.017), whereas a higher proportion of those not intending to use telehealth were from the Northern region (7.8% vs. 2.1%; *p* = 0.017). The significant regional variation observed in telehealth intention may reflect differences in healthcare infrastructure and telehealth availability across regions.

Intention to use telehealth was significantly higher among neurologists working in governmental institutions than among those in the private sector (89.8% vs. 73.4%; *p = 0*.001). Neurologists in tertiary care settings were more likely to intend to use telehealth than those in secondary care (51.3% vs. 32.8%; *p = 0*.030). Conversely, consultants demonstrated higher intention to use telehealth than specialists (36.0% vs. 21.9%; *p* = 0.006). No significant correlations were found with age, gender, years of experience, or subspecialty.

### Factors associated with intention to use telehealth

Multivariate logistic regression was used to examine some factors associated with neurologists’ intention to use telehealth- (see Table 6). Offering telephone/audio services in the healthcare organization was positively associated with intention to use telehealth services (OR = 4.84, 95% CI: 1.48–15.00; *p = 0*.009), as was preference for video-based telehealth (OR = 2.30, 95% CI: 1.06–5.00; *p = 0*.035). However, neurologists who reported having video and audio telehealth modes were less likely to use telehealth in the future (OR = 0.13, 95% CI: 0.03–0.52; *p =* 0.004). Preferring video-based telehealth over audio-only communication was independently associated with greater intention to use telehealth. Furthermore, a higher likelihood of intending to use telehealth was strongly associated with familiarity with telehealth technology (OR = 32.0, 95% CI: 8–126; *p < 0*.001). Regarding attitudinal factors, positive attitudes toward telehealth for follow-up care were strongly associated with intention to adopt telehealth (OR = 21.0, 95% CI: 7–63; *p < 0*.001). This strong association suggests that physicians’ perceptions regarding clinical suitability play a critical role in determining telehealth use. In addition, institutional availability of telephone-based services significantly predicted intention to use telehealth, emphasizing the importance of organizational infrastructure in facilitating digital healthcare delivery. Perceived safety and privacy, as well as cost reduction, were all significant factors associated with telehealth use (OR = 2.44, *p* = 0.017; OR = 2.55, *p* = 0.040, respectively).

**Table 6:** Multivariable logistic regression analysis of factors associated with intention to use telehealth among neurologists.

Factor	*B*	*p*-value	Adjusted OR (Exp[B])	CI (95%)
Telephone/audio services offered by the institution	1.57	0.009	4.84	1.48–15.00
Use of both video and audio services	−2.00	0.004	0.13	0.03–0.52
Preference for video over audio	0.83	0.035	2.30	1.06–5.00
Familiarity with telehealth technology	3.47	<0.001	32.00	8.00–126.00
Attitude domains				
Positive attitude toward telehealth for follow-up patients	3.07	<0.001	21.00	7.00–63.00
Safety and privacy concerns	0.89	0.017	2.44	1.00–5.00
Perceived cost reduction	0.93	0.040	2.55	1.04–6.25

OR, adjusted odds ratio; CI, confidence interval.

*p-value < 0.05 indicates statistical significance.

## Discussion

Telehealth is expected to play an increasingly central role in the future of healthcare services, with most healthcare systems worldwide actively integrating it (13). This study aimed to explore neurologists’ intentions and attitudes toward teleneurology by examining the availability, utilization, and perceived applicability of telehealth services within neurological practice. To the best of our knowledge, this is the first national study conducted after the lifting of COVID-19-related restrictions to evaluate multiple dimensions of teleneurology in Saudi Arabia. By examining the distribution of current telehealth services, regional gaps, and provider perspectives, this study can provide important baseline information for future teleneurology planning and policy development. This paper also assessed neurologists’ preferred methods for implementing telehealth services, which should be considered to achieve desirable outcomes in teleneurology.

The sociodemographic profile showed that most participants were younger neurologists, including residents and early-career specialists under 40 years of age, who accounted for nearly 80 percent of the sample. This demographic may reflect the near future of neurology services in Saudi Arabia. Notably, more than three-quarters of respondents showed a clear intention to adopt telehealth in their practice, regardless of age group. The absence of a clear age-related resistance suggests broad professional openness to the use of teleneurology, including among more experienced neurologists. This noticeable consistency between age distribution and years of professional experience supports the internal coherence of the sample characteristics.

In regards to the employment sector, most participants worked in the government healthcare sector, which remains the primary provider of healthcare services in Saudi Arabia. This finding may reflect the strong governmental investment in digital health infrastructure in Saudi Arabia, particularly under the national digital transformation strategy associated with Vision 2030. Although the private sector accounted for a smaller proportion of respondents, a majority of private-sector neurologists also reported a strong intention to use telehealth. According to the latest statistics available from the Ministry of Health (MOH), only 220 neurologists are currently working in the private sector (MOH, 2022). Given the expanding privatization initiatives within the Saudi healthcare system (14), teleneurology policies should be designed to support both governmental and private institutions to ensure fair access to neurological care across sectors.

Participant distribution across all healthcare levels showed that most worked in secondary and tertiary care facilities, with relatively few neurologists working in primary care. However, the intention to use telehealth was consistently higher than the refusal rate across all healthcare types. Among medical specialties, general neurologists constituted the largest group participating in this study, reflecting both trainees and practicing specialists, whereas certain subspecialties, primarily neuroimmunology, demonstrated a high willingness to adopt telehealth. These findings align with evidence suggesting that patients with chronic neurological conditions, such as multiple sclerosis, may benefit particularly from telehealth, which facilitates follow-up and monitoring (15).

Additionally, the availability of telehealth services for neurologists to use differed across governmental institutions, including MOH facilities and other government healthcare facilities, such as university hospitals. However, these differences were excluded from the analysis because insufficient information was available on the actual number of telehealth services available for each facilityAmong neurologists, the majority are found in MOH hospitals (338) and the private sector (220), while only 187 were documented in other government institutions (5). The distribution was measured based on the availability of telehealth services across institutions. Telehealth was used by neurologists in correlation with their intention to adopt telehealth services in their future work. However, this study’s findings indicated that telephone calls were the most frequently used modality for delivering healthcare via teleneurological services.

Using audio-only communication, i.e., phone calls may limit the benefits of telehealth, as it is not well-suited for performing specific neurological examinations remotely. However, with technological advances, diagnostic accuracy has improved substantially through the extensive integration of video-based examination into neurological care delivery. Several studies have demonstrated improved diagnostic accuracy and clinical outcomes when telehealth platforms incorporate video-based examinations, particularly in telestroke, epilepsy, and movement disorders (16–20). This is especially useful in rural areas, which are often characterized by limited advanced clinical facilities and relatively poor road connectivity. Telehealth has become a suitable alternative, enabling neurologists to provide care to patients in these areas (21).

Therefore, with factors such as diagnostic accuracy and effective follow-ups in mind, telemedicine is not inferior to in-person care in terms of accuracy and satisfaction, particularly for long-term treatment and follow-up of non-acute headaches, thereby reducing healthcare delivery costs (22). It is worth mentioning that virtual neurological examinations are perceived as being used more frequently by neurologists with greater experience, such as consultants, than by those with less experience (8, 12). Physical examinations can be performed in virtual video clinics, although challenges exist, such as patient condition, for example, testing reflexes, muscle tone, or detailed sensory responses. These tests are harder to perform remotely and may require an in-person visit (23). Therefore, telemedicine can support parts of the neurological examination, but cannot replace a complete in-person neurological assessment because several important clinical tests require direct physical interaction between the clinician and the patient (22).

Practitioners’ perceptions of telehealth benefits, even prior to its use, may reflect their expectations regarding telehealth (4). Interestingly, though nearly half of the neurologists in this study reported that their institutions did not offer telehealth services, the majority indicated positive intentions toward telehealth, which may reflect high acceptance even prior to using it. Additionally, neurologists who had the opportunity to use telehealth in their facilities reported a strong intention to implement this technology in their routine practice. This finding aligns with a similar study of neurologists who used telehealth during the COVID-19 pandemic; perceptions of telehealth and confidence were notably high, particularly in certain neurological subspecialties, including epilepsy and follow-up headache patients (12).

Regarding the practicality of telehealth in clinical practice, general neurology clinics were most frequently identified as suitable settings for telehealth use, particularly for screening and follow-up visits. Participants also suggested that teleneurology could facilitate triage and early assessment, enabling more efficient referral to subspecialty clinics when necessary. However, implementing these suggestions may inadvertently increase clinical investigation rates without clear benefits, as shown in previous studies comparing face-to-face visits with telemedicine visits in new outpatient neurology clinics (25, 26). This may partly reflect diagnostic uncertainty during virtual assessment, where limitations in performing a complete neurological examination may incite neurologists to order additional investigations to exclude potential abnormalities that cannot be assessed remotely (27). In pediatric neurology clinics, for example, a few participants perceived telehealth as applicable for evaluating new patients, whereas a larger number indicated that it was applicable only for follow-up visits.

Barriers to telehealth adoption vary across factors, including patients, practitioners, healthcare institutions, and the healthcare system. Patient-related barriers may play a critical role in implementing telehealth services. Limited patient awareness, levels of digital literacy, and concerns about data privacy and confidentiality may reduce patient acceptance of telehealth consultations. In addition, some patients may prefer traditional face-to-face consultations because they emphasize physical examinations in neurological diagnosis. Addressing these challenges through patient education initiatives, improved digital accessibility, and user-friendly telehealth platforms will be essential to enhancing patient engagement and maximizing the benefits of telehealth services (28–31).

From the perspectives of neurologists and healthcare organizations, limited technological competence or insufficient training among healthcare providers may reduce their confidence in using telehealth systems. A recent scoping review revealed that lack of digital skills, limited access to technology, and infrastructure limitations are among the most commonly cited barriers to telehealth adoption (32, 33). Evidence showed that adequate training and professional technical development significantly improved physicians’ confidence and attitudes toward telehealth implementation (34). Regulatory and policy-related factors may also affect the adoption of telehealth services. Telehealth delivery usually requires compliance with regulatory frameworks related to patient privacy, data protection, professional licensure, and quality assurance. In many healthcare systems, uncertainty regarding reimbursement policies, clinical governance requirements, and legal liability associated with remote consultations may discourage healthcare providers from fully adopting telehealth services. Establishing clear national policies and regulatory frameworks is therefore essential for supporting safe and sustainable telehealth implementation. Previous studies conducted in Saudi Arabia have similarly found financial sustainability, reimbursement mechanisms, and alignment with institutional priorities as key challenges affecting telemedicine adoption (28).

Technical obstacles also significantly hinder telehealth utilization. Reliable internet connectivity, appropriate hardware, and user-friendly telehealth platforms are essential for effective virtual consultations. In specialties such as neurology, where detailed clinical evaluation and visual examination are often required, poor video quality, unstable internet connections, and technical malfunctions may compromise the accuracy of remote assessments (35, 36).

In our multivariable analysis, a notable finding was the negative association between the availability of both video and audio services and the intention to use telehealth (OR = 0.13), compared to the availability of simpler modalities. This counterintuitive result may reflect the “complexity burden” often associated with early-stage implementation of comprehensive telehealth systems. Clinicians in settings attempting to deploy both video and audio simultaneously may face greater technical hurdles, connectivity issues, or workflow disruptions compared to those relying on established, simpler audio-only systems. This suggests that without adequate IT support and user-friendly interfaces, the addition of video modalities might paradoxically reduce immediate willingness to adopt the technology due to technical fatigue. Additionally, the very wide confidence intervals observed for variables such as “Familiarity” and “Attitude toward follow-up” (e.g., OR 32, OR 21) should be interpreted with caution. These wide intervals likely stem from sparse data in the reference groups (i.e., nearly all neurologists with high familiarity intended to use the service), which reduces the precision of the point estimate while still confirming the strong positive association.

Neurologists across all age groups, experience levels, and practice settings revealed a high intention to adopt telehealth. Most participants expressed willingness to incorporate telehealth into future clinical practice, including practitioners with no prior experience. This finding is important as it suggests strong readiness for the broader implementation of teleneurology in Saudi Arabia.

One important motivating factor identified in this study was cost reduction. Participants believed that cost reduction for both patients and hospitals would be desirable and achievable through telehealth. Participants perceived telehealth as an effective means of reducing patient travel, improving access for remote populations, and optimizing time efficiency for both patients and providers. These perceptions are not unique to this study; other studies have shown that telemedicine reduces geographic barriers and facilitates access to specialized neurological care at lower cost (1, 33, 34). However, concerns remain with regard to the initial capital costs of infrastructure and equipment, as well as the limitation of the robust cost-effectiveness data for teleneurology in outpatient settings.

This study has several limitations. First, the use of snowball sampling, while necessary due to the lack of a centralized registry, introduces potential selection bias. It is likely that neurologists with a pre-existing interest in technology or telehealth were more motivated to complete the survey, potentially leading to an overestimation of the prevalence of” intention to use” prevalence compared to the general population of neurologists. Second, the absence of a unified communication platform for neurologists in Saudi Arabia complicated recruitment and delayed reaching the target sample size. Third, the design used in this study examines attitudes and intentions at a single time point, which may change rapidly as telehealth services expand. Sampling bias may also be a concern due to higher participation from secondary and tertiary healthcare centers. However, the sample size is relatively large, which strengthens the validity and reliability of the findings. Given the limited national research on outpatient teleneurology, this study provides foundational evidence to guide future implementation efforts. By identifying neurologists’ attitudes, perceived barriers, and intentions, these findings can guide the development of targeted policies, training programs, and guidelines to support effective teleneurology integration in Saudi Arabia.

## Conclusion

This study provides an overview of neurologists’ perspectives on telehealth in Saudi Arabia. Most neurologists surveyed in this study expressed positive attitudes toward telehealth and a strong intention to use it in future clinical practice, particularly for follow-up care.

Several factors are associated with the intention to adopt telehealth, including positive attitudes toward follow-up care, perceived safety and privacy, cost reduction, and adequate familiarity with telehealth technology. Key challenges to the adoption of teleneurology include limited patient acceptance, insufficient training, and the absence of institutional policies guiding its use.

In sum, the study findings indicated that teleneurology might be an acceptable and feasible model of care, particularly for follow-up management across multiple neurology subspecialties. Familiarity with telehealth technology, positive perceptions of its suitability for follow-up care, and the availability of institutional audio telehealth services were independently associated with the intention to adopt telehealth services.

## Data Availability

The raw data supporting the conclusions of this article will be made available by the authors, without undue reservation.
